# Bacterial Noncoding RNAs Excised from within Protein-Coding Transcripts

**DOI:** 10.1128/mBio.01730-18

**Published:** 2018-09-25

**Authors:** Daniel Dar, Rotem Sorek

**Affiliations:** a Department of Molecular Genetics, Weizmann Institute of Science, Rehovot, Israel; University of Washington

**Keywords:** transcriptome, mRNA degradation, ncRNA, sRNA

## Abstract

Prokaryotic genomes encode a plethora of small noncoding RNAs (ncRNAs) that fine-tune the expression of specific genes. The vast majority of known bacterial ncRNAs are encoded from within intergenic regions, where their expression is controlled by promoter and terminator elements, similarly to protein-coding genes. In addition, recent studies have shown that functional ncRNAs can also be derived from gene 3′ untranslated regions (3′UTRs) via an alternative biogenesis pathway, in which the ncRNA segment is separated from the mRNA via RNase cleavage. Here, we report the detection of a large set of decay-generated noncoding RNAs (decRNAs), many of which are completely embedded within protein-coding mRNA regions rather than in the UTRs. We show that these decRNAs are “carved out” of the mRNA through the action of RNase E and that they are predicted to fold into highly stable RNA structures, similar to those of known ncRNAs. A subset of these decRNAs is predicted to interact with Hfq or ProQ or both, which act as ncRNA chaperones, and some decRNAs display evolutionarily conserved sequences and conserved expression patterns between different species. These results suggest that mRNA protein-coding regions may harbor a large set of potentially functional small RNAs.

## Abstract (precis)

Bacteria and archaea utilize regulatory small noncoding RNAs (ncRNAs) to control the expression of specific genetic programs. These ncRNAs are almost exclusively encoded within intergenic regions and are independently transcribed. Here, we report on a large set ncRNAs that are “carved out” from within the protein-coding regions of Escherichia coli mRNAs by cellular RNases. These protected mRNA fragments fold into energetically stable RNA structures, reminiscent of those of intergenic regulatory ncRNAs. In addition, a subset of these ncRNAs coprecipitate with the major ncRNA chaperones Hfq and ProQ and display evolutionarily conserved sequences and conserved expression patterns between different bacterial species. Our data suggest that protein-coding genes can potentially act as a reservoir of regulatory ncRNAs.

## OBSERVATION

Small noncoding RNAs (ncRNAs) control gene expression in prokaryotes, regulating processes such as nutrient acquisition, stress responses, virulence, and biofilm formation (1–5). Generally, ncRNAs tune the expression of their target gene via direct base pairing with mRNA regions, leading to altered translation efficiency or mRNA stability (2, 4, 6). Regulation via base pairing is often dependent on specialized proteinaceous chaperones, such as Hfq or ProQ, which act as matchmakers between ncRNAs and target mRNAs (7–9). Alternatively, ncRNAs can interact with and control the activity of RNA-binding proteins such as CsrA or RNA polymerase, which are regulated by binding the CsrB/C ncRNAs and the 6S ncRNAs, respectively (1).

Prokaryotic genomes can harbor large ncRNAs and *cis*-encoded antisense transcript repertoires (1, 10, 11). Previously described ncRNAs are almost exclusively encoded within intergenic regions, including gene 3′ untranslated regions (3′UTRs) (2), where their expression is controlled via promoter and terminator elements, akin to that of protein-coding genes. However, recent studies indicate that ncRNAs can also be derived from gene 3′UTRs, via an alternative RNase-dependent biogenesis pathway (12–15). For example, the *cpxP* membrane stress response chaperone is transcribed into an mRNA containing a conserved ∼60-nucleotide (nt) 3′UTR, which can independently act as an Hfq-dependent ncRNA. This ncRNA, CpxQ, is separated from the mRNA by RNase E, enabling its activity as a regulator of multiple genes (12). Here, we report that bacterial ncRNAs can also be “carved out” of protein-coding regions rather than 3′UTRs or 5′UTRs during the process of mRNA decay.

### Systematic discovery of mRNA-derived decay-generated noncoding RNAs (decRNAs) in Escherichia coli.

Recently, we used RNA sequencing (RNA-seq) to track mRNA decay dynamics in bacteria. By exposing Escherichia coli to rifampicin, a transcription initiation inhibitor, and then sampling the cultures at ∼1-min intervals following transcription inhibition, we recorded the decay rates of expressed genes at high temporal resolution (16). We reported many cases where genes corresponding to the same multigene polycistronic transcript underwent differential decay such that some pieces of the transcript underwent rapid decay whereas others were stabilized (16).

**FIG 1:**
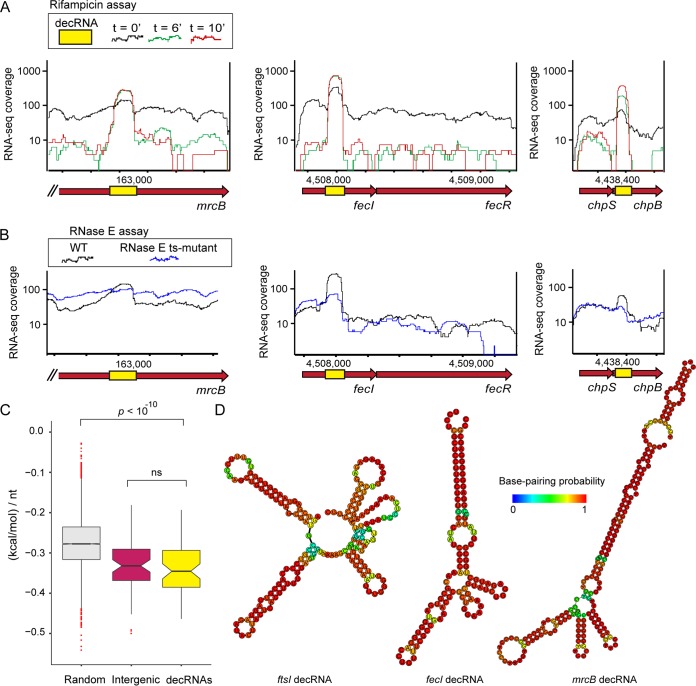
Detection of ncRNAs generated from within protein-coding sequences. (A) Examples of decRNAs (yellow boxes) located within E. coli protein-coding sequences (red arrows). RNA-seq coverage data from a rifampicin mRNA decay assay (16) are shown for the steady-state time point (black, *t* = 0) and at two time points (indicated with green and red) following inhibition of transcription initiation by rifampicin. RNA-seq coverage was normalized by the number of mapped reads in each library. (B) RNA-seq experiments performed with the WT strain or a temperature-sensitive RNase E mutant, both incubated in the restrictive temperature of 44°C for 10 min. Data representing RNA-seq coverage of WT and of mutant RNase E are shown in black and blue, respectively. (C) Box plot showing predicted RNA structure stability for decRNAs described in this study (yellow; *n* = 58), previously described E. coli ncRNAs encoded in intergenic regions (purple, *n* = 63), and randomly selected genomic sequences of similar lengths (gray, *n* = 10,000). The predicted fold stability values were normalized by the ncRNA/segment length (in kilocalories per mole per nucleotide [nt]). Outliers are depicted as red dots. The distributions were compared using a two-sided Wilcoxon rank sum test, and significant *P* values are shown in the figure. (D) Examples of predicted RNA structures for three representative decRNAs (Table S1). The color scheme shows the calculated base-pairing probability for the predicted structures (21).

In this study, we reanalyzed our published mRNA decay experiments (16) in search of subgene differential decay patterns. Specifically, we searched for mRNAs (5′UTR and 3′UTR included) that decay in a nonuniform manner, leaving behind short, highly stabilized segments that potentially function as ncRNAs, following mRNA degradation (Fig. 1A). While most E. coli mRNAs displayed uniform decay profiles across the entire transcript body, our analysis recovered 58 mRNA subsegments, 80 to 300 nt in size (average of ∼160 nt), that were preferentially stabilized compared with the rest of the transcript (Fig. 1A; see also Table S1 in the supplemental material). In these cases, the stabilized mRNA segment decays at a slower rate and thus accumulates to higher steady-state levels than its original host mRNAs (Fig. 1A; see also Table  S1). This set includes the *sroC* regulatory ncRNA, which is known to be cleaved out of the *gltI* gene 3′UTR (17) (Table S1). Over 82% (48/58) of the RNAs in our set significantly overlapped the protein-coding sequence of the mRNA (i.e., more than half of the stabilized mRNA segment overlapped the coding sequence). In 36% (21/58) of the cases, the stabilized RNA was entirely carried within the coding sequence (Fig. 1; see also Table S1).

10.1128/mBio.01730-18.5 TABLE S1 List of decRNAs discovered in this study. The list represents 58 decRNAs detected in this study. The table contains information about the host gene, the percentage of overlap of the gene coding region, the decRNA genomic coordinates and sequence, and the predicted RNA folding pattern and stability. Download Table S1, XLSX file, 0.02 MB. Copyright © 2018 Dar and Sorek. 2018 Dar and Sorek This content is distributed under the terms of the Creative Commons Attribution 4.0 International license.

To independently validate that these stabilized RNA fragments are liberated from mRNAs through RNase activity, we analyzed total RNA from E. coli harboring a temperature-sensitive mutation in RNase E (18). In these cells, RNase E (the major endoribonuclease in E. coli) is functional at 30°C but is inactive at 44°C (19). We sequenced the RNAs of the wild-type (WT) strain and the mutant strain following a brief 10-min incubation at the restrictive temperature of 44°C (16). We found that the vast majority of stabilized RNAs that we detected showed significantly diminished abundance as a result of RNase E inactivation, confirming their dependence on RNase E for their biogenesis (Fig. 1B; see also Fig. S1 in the supplemental material) (*P  < *10^−9^ [Wilcoxon]).

10.1128/mBio.01730-18.1 FIG S1 decRNA generation is dependent on RNase E activity. Data are presented as box plot distributions showing the fold differences in expression between decRNAs and their respective host genes (decRNA expression/host gene expression), calculated for all decRNAs reported in this study (Table S1). Values were calculated from RNA-seq data taken from the WT (gray) and temperature-sensitive RNase E mutant (blue) strains, grown at the nonpermissive temperature of 44°C, where the RNase E mutant is deactivated but the WT RNase E remains functional. Outliers are shown as black dots. The distributions were compared using a two-sided Wilcoxon rank sum test, and significant *P* values are shown in the figure. Download FIG S1, TIF file, 0.1 MB. Copyright © 2018 Dar and Sorek. 2018 Dar and Sorek This content is distributed under the terms of the Creative Commons Attribution 4.0 International license.

Analyzing the sequences and predicted RNA structures of these stabilized mRNA fragments showed that they displayed elaborate folding patterns that were significantly more energetically stable than those generated by random genomic segments of similar sizes (Fig. 1C and D; *P  < *10^−10^ [Wilcoxon]) and of stability similar to that of known, intergenic ncRNAs (Fig. 1C). In addition to their highly stable structures, these stabilized RNA species seem to lack canonical ribosome binding sites or convincing open reading frames (ORFs). Together, these results reveal a large set of short RNAs in E. coli that are “carved out” from within protein-coding mRNA regions.

### Interaction of putative decRNAs with Hfq and ProQ.

The regulatory activity of ncRNAs in E. coli is often dependent on physical association with Hfq (7) or ProQ (8, 9), both of which act as chaperones that increase the efficiency of the interaction between ncRNAs and their target mRNAs. To assess whether the decRNAs described above potentially interact with these RNA chaperones, we reanalyzed published Hfq and ProQ coimmunoprecipitation (co-IP) experiments followed by RNA sequencing (RNA-seq) (8, 20). We found that 31% (18/58) of the decRNAs in our set are predicted to interact with Hfq or ProQ under the tested experimental conditions and that some might interact with both factors (Fig. S2 and S3; see also Table S2). Similar enrichment values were detected for known regulatory ncRNAs. Nearly all the Hfq- or ProQ-associated decRNAs in our set overlapped the 3′UTR or 5′UTR of their host mRNA (Tables S1 and S2). Nonetheless, many of these decRNAs substantially overlapped the protein-coding region, and the observed ProQ or Hfq enrichment was often detected with the protein-coding region of the decRNA rather than the UTR (Fig. S2 and S3). Together, these data indicate that a substantial subset of the decRNAs detected in our analysis likely associate with the Hfq and/or ProQ ncRNA chaperones.

10.1128/mBio.01730-18.6 TABLE S2 List of decRNAs predicted to interact with ProQ or with Hfq. Detected decRNAs that potentially interact with ProQ or Hfq are indicated along with their enrichment values. Download Table S2, XLSX file, 0.01 MB. Copyright © 2018 Dar and Sorek. 2018 Dar and Sorek This content is distributed under the terms of the Creative Commons Attribution 4.0 International license.

10.1128/mBio.01730-18.2 FIG S2 Putative decRNA interactions with ProQ. Interactions between decRNAs and ProQ were evaluated using recently published data from ProQ immunoprecipitation (IP) followed by RNA-sequencing analysis (8). RNA-seq coverage plots of lysate and IP samples are shown as black and green lines, respectively. RNA-seq coverage was normalized by the number of mapped reads in each library. The decRNAs are depicted as yellow boxes. Panels A to C show three putative decRNA-ProQ interactions with two tracks: control IP in a WT ProQ strain and (below) the ProQ-3xFLAG experiment (ProQ-IP). The final enrichment value is indicated in the figure. Download FIG S2, TIF file, 0.6 MB. Copyright © 2018 Dar and Sorek. 2018 Dar and Sorek This content is distributed under the terms of the Creative Commons Attribution 4.0 International license.

10.1128/mBio.01730-18.3 FIG S3 Putative decRNA interactions with Hfq. Interactions between decRNAs and Hfq were evaluated using recently published data from Hfq immunoprecipitation followed by RNA sequencing (21). IP sample coverage data are shown in black. decRNAs are depicted as yellow boxes. Panels A to C show three putative decRNA-Hfq interactions. Download FIG S3, TIF file, 0.3 MB. Copyright © 2018 Dar and Sorek. 2018 Dar and Sorek This content is distributed under the terms of the Creative Commons Attribution 4.0 International license.

Since many decRNAs in our set are completely embedded within protein-coding regions, their 3′ termini do not generally end at an intrinsic terminator motif (stem-loop followed by a U-tract), which is one of the major elements required for interaction with Hfq (7) and possibly a significant determinant for interaction with ProQ (8, 9). Nonetheless, our analysis described above revealed several potential Hfq- or ProQ-binding decRNAs that are entirely or almost entirely embedded within the protein-coding region of the mRNA (Fig. S2 and S3; see also Tables S1 and S2). For example, the *ftsI*-decRNA, embedded within penicillin-binding protein-3, is predicted to bind both Hfq and ProQ (Fig. S2 and S3); the *anmK*-decRNA, located at the center of anhydro-N-acetylmuramic acid kinase, specifically associated with Hfq; and the *pncB*-decRNA, present within the nicotinate phosphoribosyltransferase protein, was highly enriched in the ProQ pulldown experiments (Table S2).

**FIG 2:**
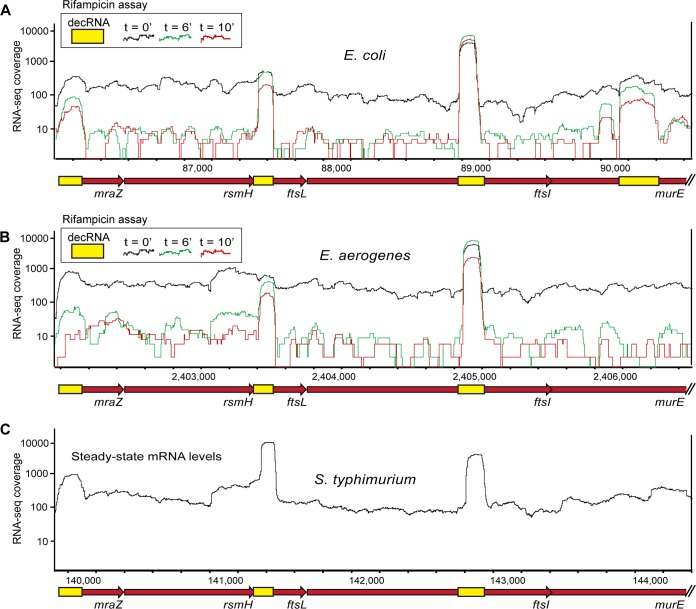
Conserved decRNAs expression signatures in evolutionarily diverged bacteria. (A) The E. coli *mraZ-rsmH-ftsL-ftsI-murE*-containing operon, encoding four predicted decRNAs (yellow boxes). RNA-seq coverage is shown for the steady-state time point (black, *t* = 0) and for two time points (indicated in green and red) following rifampicin treatment. (B) The E. aerogenes *mraZ-rsmH-ftsL-ftsI-murE*-containing operon with an overlay of the same time points taken from an identically performed rifampicin RNA-decay assay (16). (C) The S. Typhimurium *mraZ-rsmH-ftsL-ftsI-murE*-containing operon, presented with the steady-state RNA-seq coverage indicated in black (16). For panels B and C, RNA-seq coverage was normalized by the number of mapped reads in each compared library.

A closer examination of the *ftsI*-decRNA revealed that it is part of the *mraZ-rsmH-ftsL-ftsI-murE*-containing operon, which includes 3 additional decRNAs, encoded within *mraZ*, *ftsL*, and *murE* (Fig. 2; see also Table S1). The *ftsI*-decRNA is an abundant 173-nt fragment derived from the middle of the essential *ftsI* gene, present at an abundance that is ∼45-fold higher than that of *ftsI* mRNA, at the steady state. The *ftsI*-decRNA forms an elaborate structure that contains energetically stable stem-loops at both termini, possibly explaining its resistance to RNase digestion (16, 18) (Fig. 1D; see also Table S1). Comparison of the *ftsI* gene nucleotide sequences in bacteria belonging to the *Enterobacteriaceae* family showed that the *ftsI*-decRNA is considerably more conserved than the remaining *ftsI* coding sequence (Fig. S4). While most of the *ftsI* coding sequence shows a typical conservation pattern of higher rate of mutations at synonymous codon positions, the *ftsI*-decRNA region was largely conserved throughput its sequence, with less of a tendency for synonymous mutations (Fig. S4).

10.1128/mBio.01730-18.4 FIG S4 Evolutionary conservation of the *ftsI* gene coding sequence in enterobacteria. Multiple-sequence alignment is shown for the nucleotide sequences of the ORF of the *ftsI* gene sampled from representative enterobacteria. Stars represent 100% conservation in the 9 species in the alignment. The *ftsI*-decRNA region is marked in yellow. Download FIG S4, TIF file, 0.9 MB. Copyright © 2018 Dar and Sorek. 2018 Dar and Sorek This content is distributed under the terms of the Creative Commons Attribution 4.0 International license.

Examining the transcriptomes of two additional enterobacteria, Enterobacter aerogenes and Salmonella enterica serovar Typhimurium, demonstrated that the *ftsI*-decRNA, as well as the *mraZ*- and *ftsL*-derived decRNAs encoded in the same operon, showed clear patterns of stabilization and abundance similar to those seen with E. coli (Fig. 2). The sequence conservation of these decRNAs, as well as the observed conservation in their expression across different species, strongly suggests that these decRNAs have a conserved function beyond their capacity as part of the protein-coding genes from which they are generated.

### Discussion.

We describe a set of decRNAs that appear to be “carved out” from within protein-coding mRNA regions. These RNAs are detected across multiple RNA-seq data sets, including those published by other groups (8, 20). While the stable RNA structures of decRNAs can explain their RNase resistance (18), it is also possible that they are protected from RNase E through base-pairing interactions with other Hfq- or ProQ-dependent ncRNAs.

Intergenically encoded ncRNAs have been shown to play important roles in diverse biological processes, and yet their discovery and analysis continue to produce insights into microbial biology (1). The multilayered coding strategy of decRNA-harboring genes uses the same genetic information to produce a functional protein and a decRNA. Thus, it is possible that microbial genomes may actually encode substantially more ncRNAs than was previously known. While the RNase repertoire can differ significantly between bacterial lineages, it is possible that decRNAs could be generated in additional organisms beyond enterobacteria.

While we have not described a specific function for decRNAs, the notable evolutionary conservation and abundance of several of these, as well as the coimmunoprecipitation with Hfq and ProQ, suggest that at least some decRNAs likely play biologically meaningful roles. The nature of these biological roles, however, remains to be elucidated.

### Methods. (i) Transcriptome-wide mRNA decay and RNase mutant experiments.

Previously published rifampicin RNA-decay time-series experiments were taken from reference 16. Briefly, in these published experiments E. coli or E. aerogenes overnight cultures were diluted 1:100 into 25 ml fresh LB media and incubated at 37°C until they reached an optical density at 600 nm (OD_600_) of 0.5. Rifampicin was added to the cultures to reach a final concentration of 500 µg/ml, and samples were collected at different time points as noted previously (16). For the RNase E experiments, WT E. coli and the temperature-sensitive RNase E mutant were grown at the permissive temperature of 30°C overnight in LB and were diluted 1:100 in fresh LB media the next day. The cultures were then grown at 30°C until an OD_600_ of 0.5 was reached and were then incubated at 44°C for 10 min. The samples were mixed with ice-cold stop solution (90% ethanol and 10% saturated phenol) to deactivate cellular processes and were then collected via centrifugation at 4°C. RNA extraction and whole-transcriptome sequencing were performed as described in reference 16.

### (ii) RNA sequencing and read mapping.

Libraries for the RNase E experiment were sequenced on the Illumina Nextseq 500 platform in paired-end mode for this study. Sequencing reads generated from the RNase E experiment or previous data downloaded from reference 16 were mapped to the CP009273, NC_016810.1, and NC_015663.1 RefSeq genomes (for E. coli, S. Typhimurium, and E. aerogenes, respectively), using NovoAlign (Novocraft) V3.02.02 with default parameters, discarding reads that were nonuniquely mapped as previously described (16).

### (iii) decRNA detection and analysis.

E. coli gene annotations were recovered from NCBI and the 5′ and 3′ UTRs of every expressed gene were mapped using 5′-end sequencing and term-seq data when possible, as was recently described (16). RNA-seq data from the rifampicin experiment were used to generate maps showing coverage corresponding to the number of reads per nucleotide for each time point in each experiment, in triplicate. For each gene, the median (reads per nucleotide) was calculated using genomic coordinates that included the entire ORF and any 5′UTR or 3′UTR regions that were mapped (16). Genes covered by a median less than 5 reads/nt in the time zero samples (prior to rifampicin addition) were not considered in this analysis. Differentially decaying subgene segments were recovered using a sliding-window-based approach. For each experiment, a 100-nt window was moved in 1-nt intervals across each expressed gene. In each step, the average and standard deviation of the coverage in the window were calculated and compared with the coverage of the downstream nucleotide. Gene segments composed of >40 nt which displayed significantly increased coverage (greater than 4 standard deviations) were identified. Segments with >2-fold-higher expression than the median expression of the gene, at time zero, and that showed an increase of at least 3-fold in relative abundance during the decay time course were collected for each of the rifampicin experiment replicates. The coordinates of differentially decaying segments that passed these thresholds in at least 2 replicates were intersected to identify regions that consistently appeared stabilized. In cases where nearby 3′- or 5′-end data were available, the exact decRNA coordinates were corrected to match the mapped termini (16).

Data corresponding to the level of RNA structure stability per nucleotide (expressed in kilocalories per mole per nucleotide) were calculated using the RNA fold energetic stability score (21) divided by the length of the input sequence. The stability per nucleotide distribution of annotated ncRNAs encoded in intergenic regions (*n* = 63) downloaded from NCBI and that of decRNAs (*n* = 58) and random E. coli genomic sequences (*n* = 10,000) were compared using a two-sided Wilcoxon rank sum test. The length of the random sequence set was selected with a length distribution representative of the 121 ncRNAs described above.

Putative interactions between decRNAs and Hfq or ProQ were determined using previously published data (8, 20). In each case, the data were downloaded and mapped to the reference genome as described above. Putative Hfq interactions were determined by counting mean reads per nucleotide in the Hfq pulldown experiment. Mean coverage of greater than 30 reads/nt was considered representative of a putative interaction with Hfq. Putative ProQ-dependent interactions were assessed by comparing the level of enrichment of each decRNA in the ProQ-3xFLAG experiment to that of each decRNA in the WT ProQ experiment using previously published data (8). For each decRNA, the mean coverage in the IP and lysate samples was calculated and the IP/lysate signal ratio was determined after normalization using the total number of mapped reads per library. The signal ratios were independently calculated for the ProQ-3xFLAG experiments and the ProQ-WT experiments, and the final enrichment value was calculated as their ratio (signal in 3xFLAG experiments/signal in the WT experiments), as described in reference 8. Cases in which the decRNA was covered by a mean (reads per nucleotide) value of greater than 20 and in which the enrichment value was greater than 2 were assigned as putative ProQ interactions. Identical calculations were made with the 63 intergenic ncRNAs downloaded from NCBI, and similar enrichment values were found in most known cases. For example, the putative ProQ-binding decRNAs had an average enrichment level of 4.1, similar to the enrichment seen with validated proQ targets, such as *sibA*, *sraB*, and *istR* (8).

### Accession number(s).

The sequencing data were deposited in the European Nucleotide Archive (ENA) under accession no. PRJEB27518.
