# Supplementary figures and images for: Bacterial Noncoding RNAs Excised from within Protein-Coding Transcripts
Source: mBio. 2018 Sep 25;9(5):e01730-18. doi: 10.1128/mBio.01730-18 (PMC6156199; doi:10.1128/mBio.01730-18)

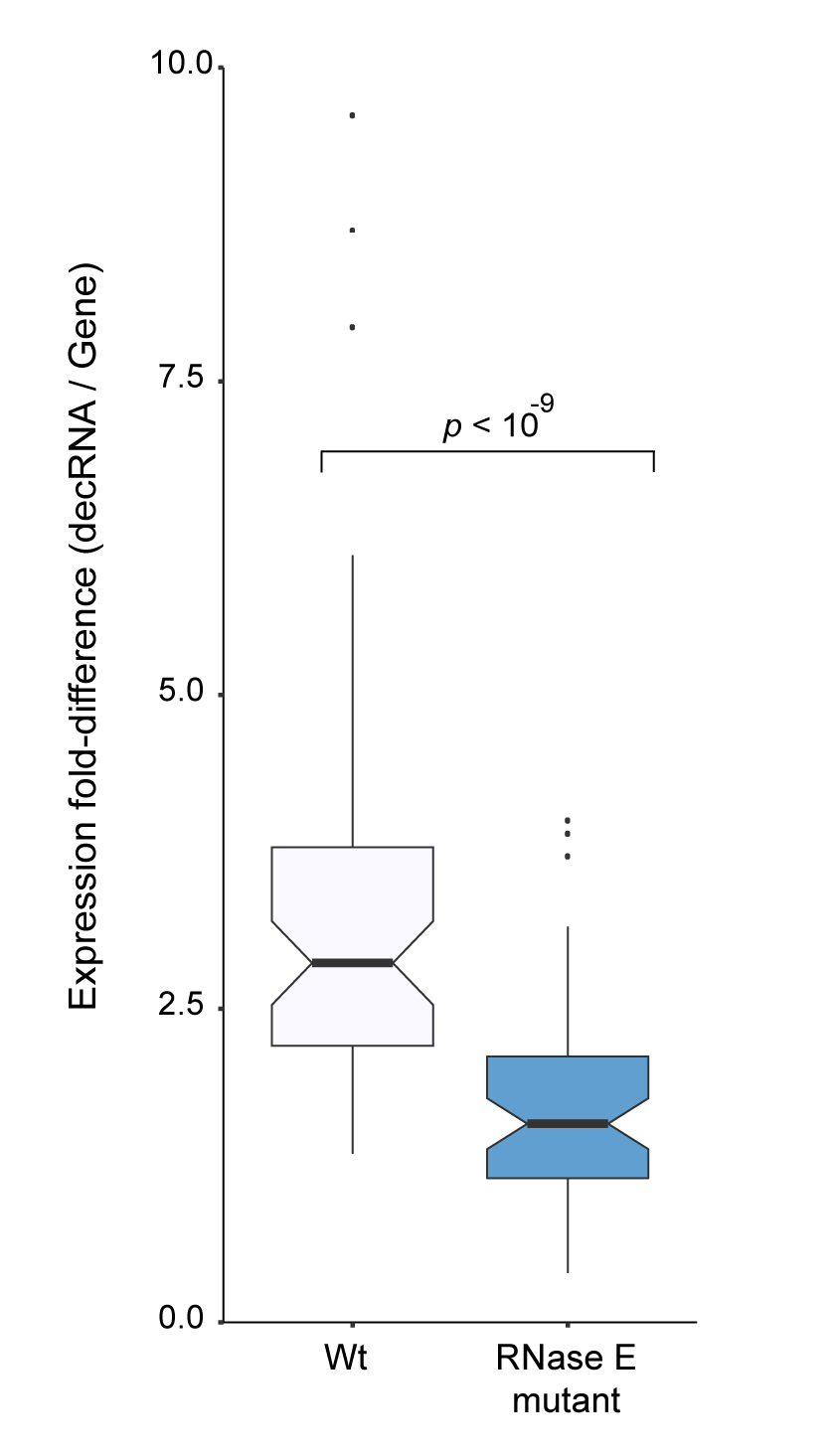

Supplement: FIG S1 [file mbo004184071sf1.tif]

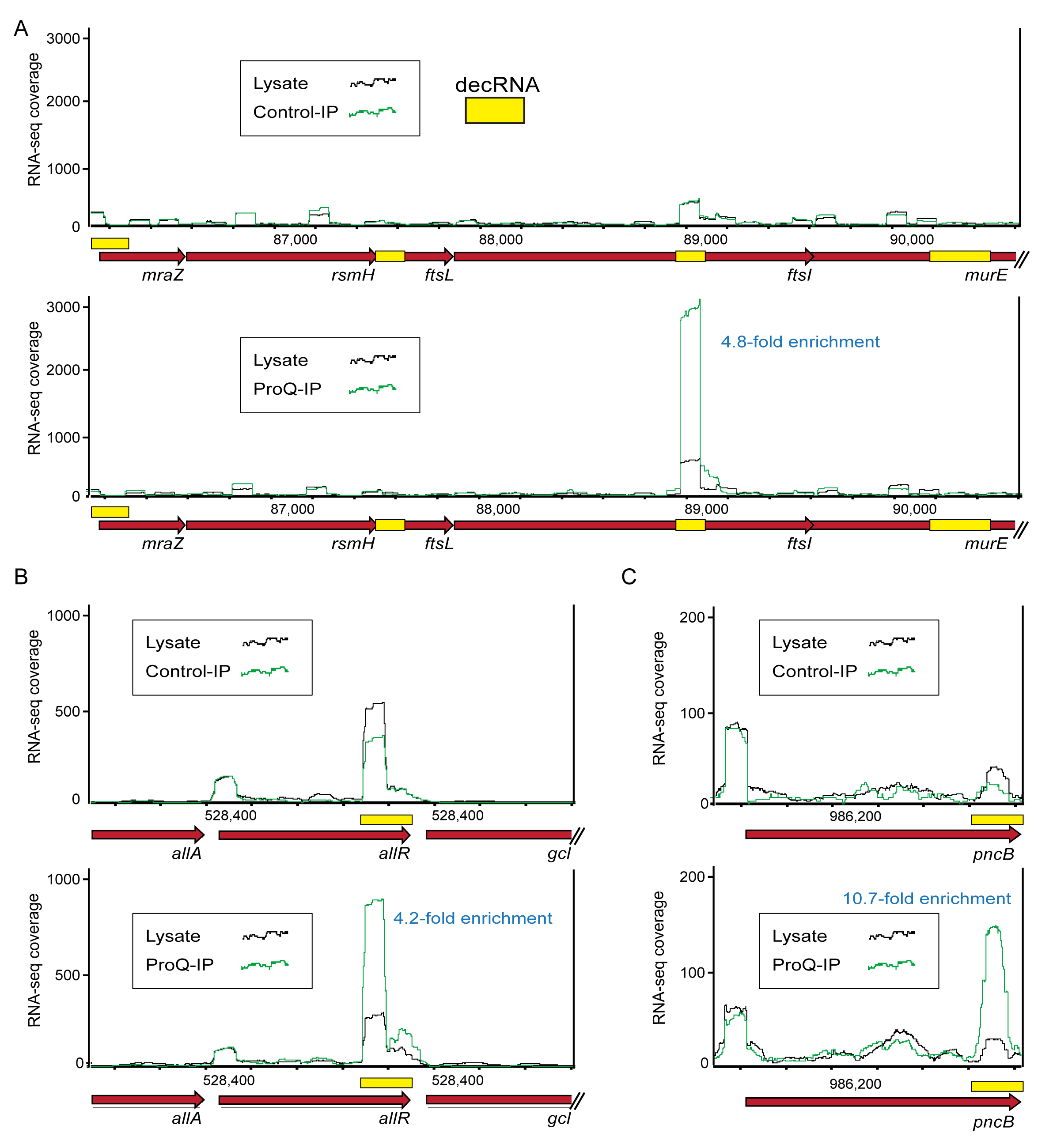

Supplement: FIG S2 [file mbo004184071sf2.tif]

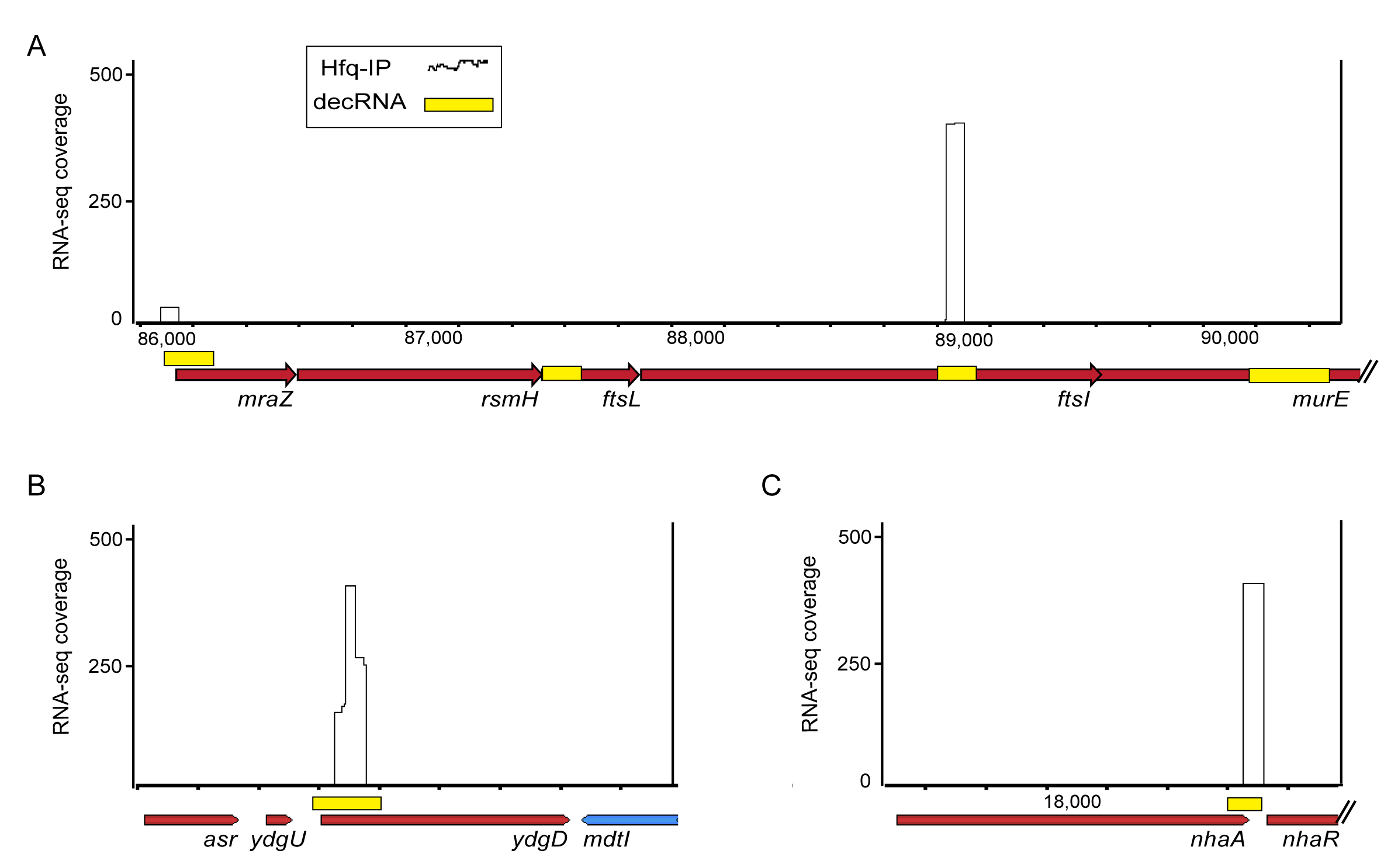

Supplement: FIG S3 [file mbo004184071sf3.tif]

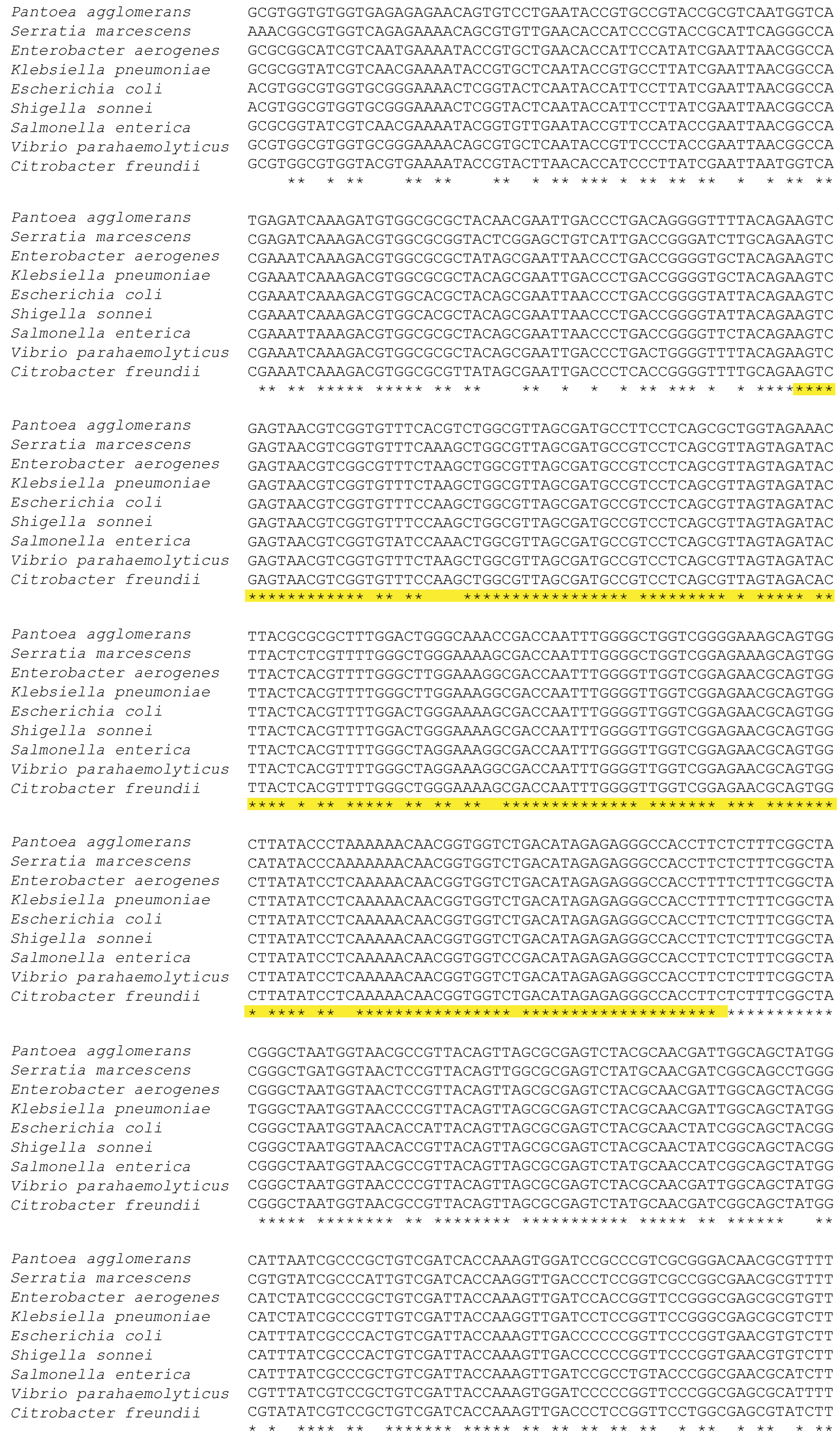

Supplement: FIG S4 [file mbo004184071sf4.tif]
